# Supplementary material for: Quantifying cancer- and drug-induced changes in Shannon information capacity of RTK signaling
Source: bioRxiv. 2025 May 5:2025.04.30.651439. Preprint. [Version 1] doi: 10.1101/2025.04.30.651439 (PMC12247869; doi:10.1101/2025.04.30.651439)
Supplement: 1 [file NIHPP2025.04.30.651439V1-supplement-1.pdf]

bioRxiv preprint doi: https://doi.org/10.1101/2025.04.30.651439; this version posted May 5, 2025. The copyright holder for this preprint (which was not certified by peer review) is the author/funder, who has granted bioRxiv a license to display the preprint in perpetuity. It is made available under aCC-BY 4.0 International license.

Appendix

Quantifying cancer- and drug-induced changes in Shannon information capacity of RTK signaling

Paweł Nałęcz-Jawecki[1]*, Lee Roth[2]*, Frederic Grabowski[1], Sunnie Li[2], Marek Kochańczyk[1], Lukasz J. Bugaj[2✉], Tomasz Lipniacki[1,3✉]

[1] Institute of Fundamental Technological Research, Polish Academy of Sciences, Warsaw, Poland
[2] Department of Bioengineering, University of Pennsylvania, Philadelphia, PA, 19104, USA
[3] Department of Statistics, Rice University, TX, 77251, USA

* These authors contributed equally.

✉ Corresponding authors: tlipnia@ippt.pan.pl (T.L.), bugaj@seas.upenn.edu (L.J.B.)

This file contains the following material:

- **Fig S1. Cell preselection based on optoFGFR receptor level.**
- **Fig S2. Response amplitude.**
- **Fig S3. Comparison of ERK-KTR translocation in STE-1 cells with and without ALKi.**
- **Fig S4. Dip amplitude.**
- **Fig S5. Leave-one-out network evaluation.**
- **Fig S6. Mean logit Bayesian update u_{Bayes} as a function of $last_k$ and $interval_k$.**
- **Fig S7. Imputation of responses after intervals not present in the dataset.**
- **Fig S8. Input interval distribution optimized without regularization.**

bioRxiv preprint doi: https://doi.org/10.1101/2025.04.30.651439; this version posted May 5, 2025. The copyright holder for this preprint (which was not certified by peer review) is the author/funder, who has granted bioRxiv a license to display the preprint in perpetuity. It is made available under aCC-BY 4.0 International license.

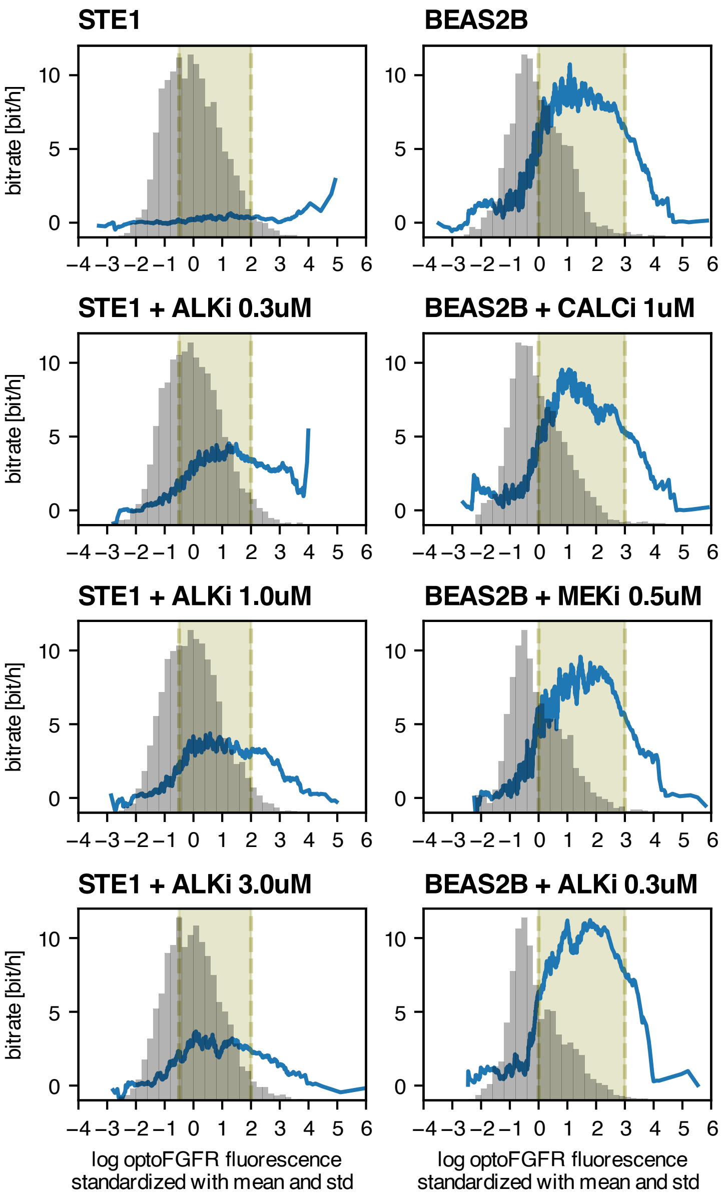

Fig S1. Cell preselection based on optoFGFR receptor level. Average bitrate in individual cells as a function of mean optoFGFR fluorescence (rolling mean over single-cell trajectories; trajectories in each rolling window have a combined duration equal to 100 times the experiment's duration), superimposed over the histograms of mean optoFGFR fluorescence in individual cells. OptoFGFR levels were log-transformed and standardized within each replicate separately, then pooled by cell line and condition. Cells in the highlighted range were used in further analysis.

bioRxiv preprint doi: https://doi.org/10.1101/2025.04.30.651439; this version posted May 5, 2025. The copyright holder for this preprint (which was not certified by peer review) is the author/funder, who has granted bioRxiv a license to display the preprint in perpetuity. It is made available under aCC-BY 4.0 International license.

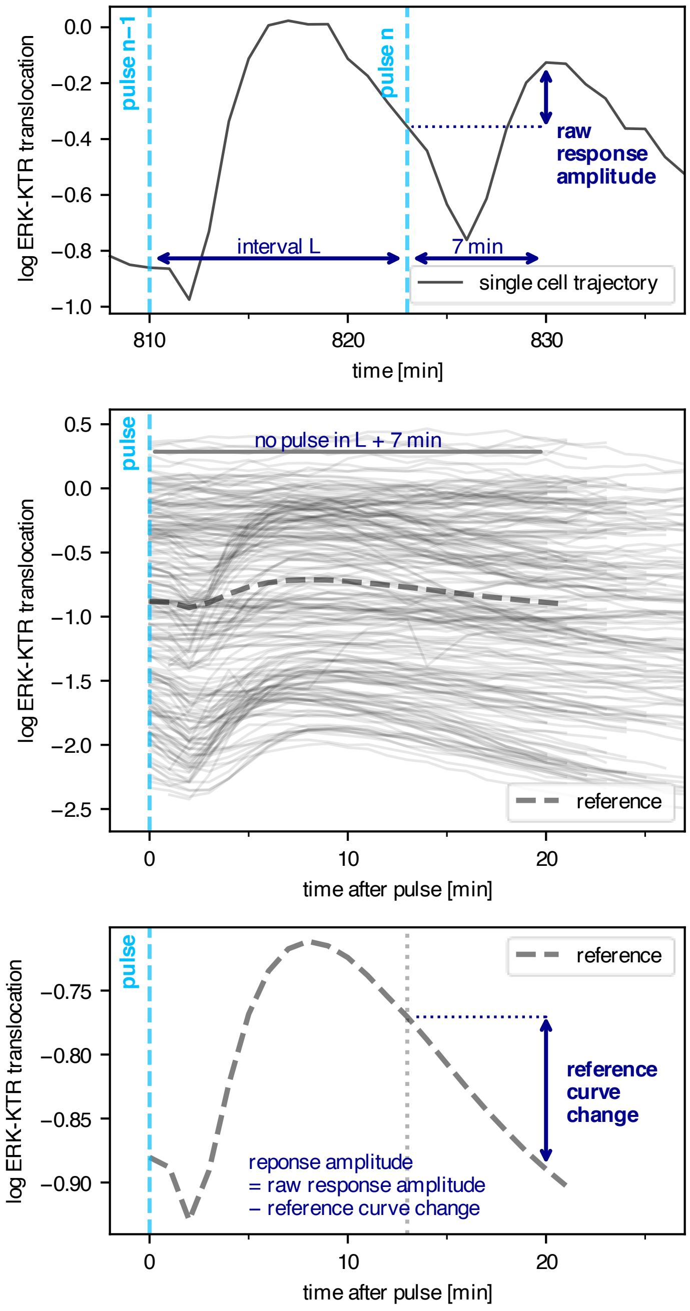

Fig S2. Response amplitude.

(A) To compute the response amplitude to light pulse *n*, which occurred time *L* after pulse ($n - 1$), we first calculated the raw response amplitude as the difference between the ERK-KTR trajectory at pulse and 7 min after.

(B) We constructed a reference trajectory by averaging the ERK-KTR trajectories after all pulses not followed by another pulse within the next L + 7 min.

(C) We computed the change in the reference trajectory between time L and (L + 7 min) after the pulse. The response amplitude was calculated by subtracting the obtained value from the raw response amplitude.

bioRxiv preprint doi: https://doi.org/10.1101/2025.04.30.651439; this version posted May 5, 2025. The copyright holder for this preprint (which was not certified by peer review) is the author/funder, who has granted bioRxiv a license to display the preprint in perpetuity. It is made available under aCC-BY 4.0 International license.

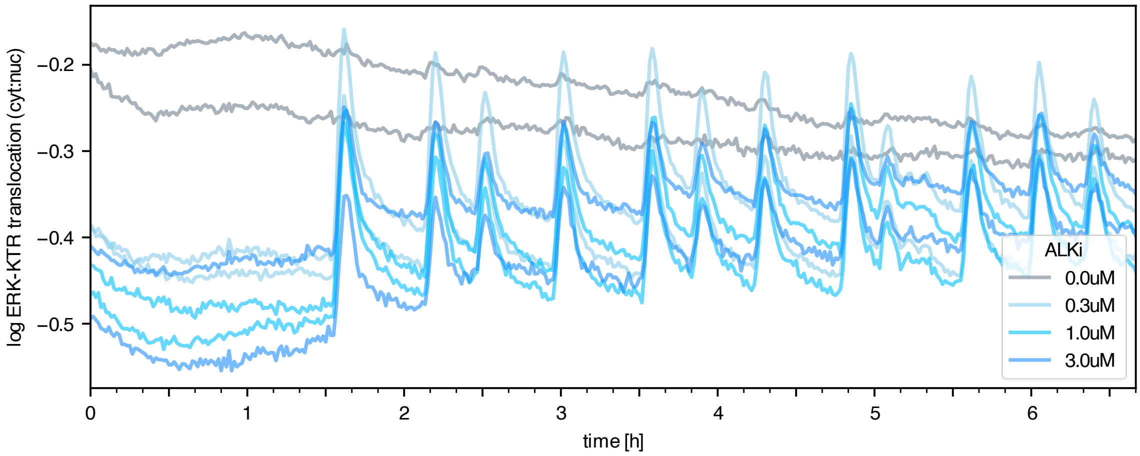

Fig S3. Comparison of ERK-KTR translocation in STE-1 cells with and without ALKi. Each line represents the ERK-KTR trajectory averaged over all cells in a single technical replicate. Note that in this figure (and only here), the ERK-KTR trajectory is computed as the log-ratio of cytoplasmic and nuclear ERK-KTR fluorescence, which allows for comparison between conditions and replicates.

bioRxiv preprint doi: https://doi.org/10.1101/2025.04.30.651439; this version posted May 5, 2025. The copyright holder for this preprint (which was not certified by peer review) is the author/funder, who has granted bioRxiv a license to display the preprint in perpetuity. It is made available under aCC-BY 4.0 International license.

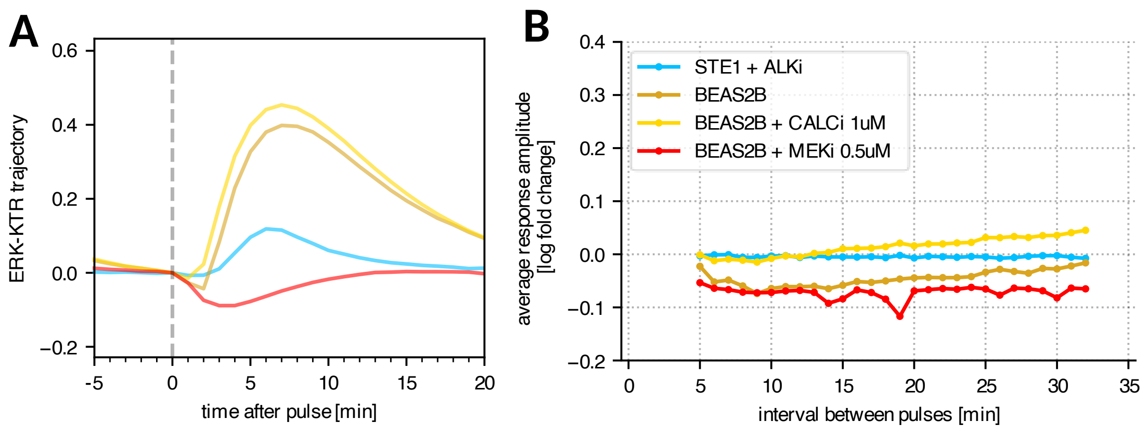

Fig S4. Dip amplitude.

(A) Population-average ERK-KTR response to a sample stimulation pulse. Color lines correspond to cell lines and conditions, as shown in panel B. The ERK-KTR dip is visible only in BEAS-2B cells with no inhibitor and with MEK inhibitor.

(B) Average response amplitude 2 min after light pulse as a function of time since the previous light pulse.

bioRxiv preprint doi: https://doi.org/10.1101/2025.04.30.651439; this version posted May 5, 2025. The copyright holder for this preprint (which was not certified by peer review) is the author/funder, who has granted bioRxiv a license to display the preprint in perpetuity. It is made available under aCC-BY 4.0 International license.

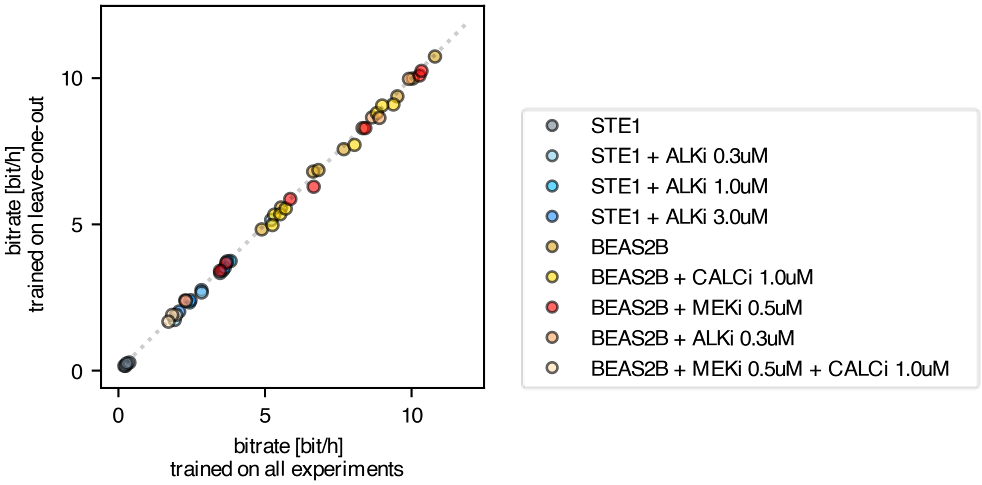

Fig S5. Leave-one-out network evaluation. Average bitrate in particular replicates, computed based on predictions by MLP trained on all conditions and replicates (as used throughout the paper; x-axis), and on all conditions and replicates except for the currently evaluated replicate (y-axis). Dotted line denotes the identity line.

bioRxiv preprint doi: https://doi.org/10.1101/2025.04.30.651439; this version posted May 5, 2025. The copyright holder for this preprint (which was not certified by peer review) is the author/funder, who has granted bioRxiv a license to display the preprint in perpetuity. It is made available under aCC-BY 4.0 International license.

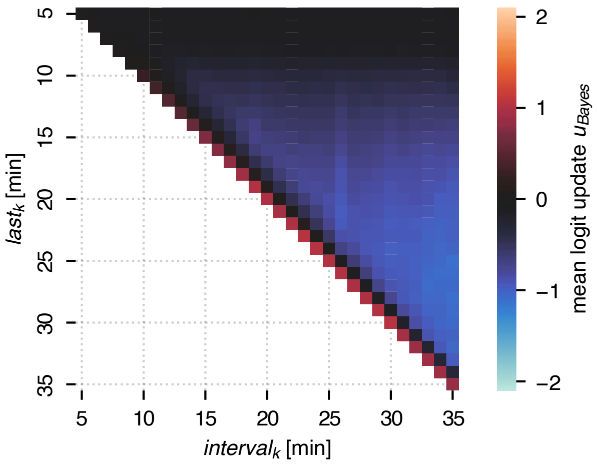

Fig S6. Mean logit Bayesian update u_{Bayes} as a function of $last_k$ and $interval_k$, evaluated on STE1 cells with ALKi. The diagonal ($last_k = interval_k$) corresponds to timepoints in which a pulse occurred; the u_{Bayes} values on the diagonal are presented in Fig 4B.

bioRxiv preprint doi: https://doi.org/10.1101/2025.04.30.651439; this version posted May 5, 2025. The copyright holder for this preprint (which was not certified by peer review) is the author/funder, who has granted bioRxiv a license to display the preprint in perpetuity. It is made available under aCC-BY 4.0 International license.

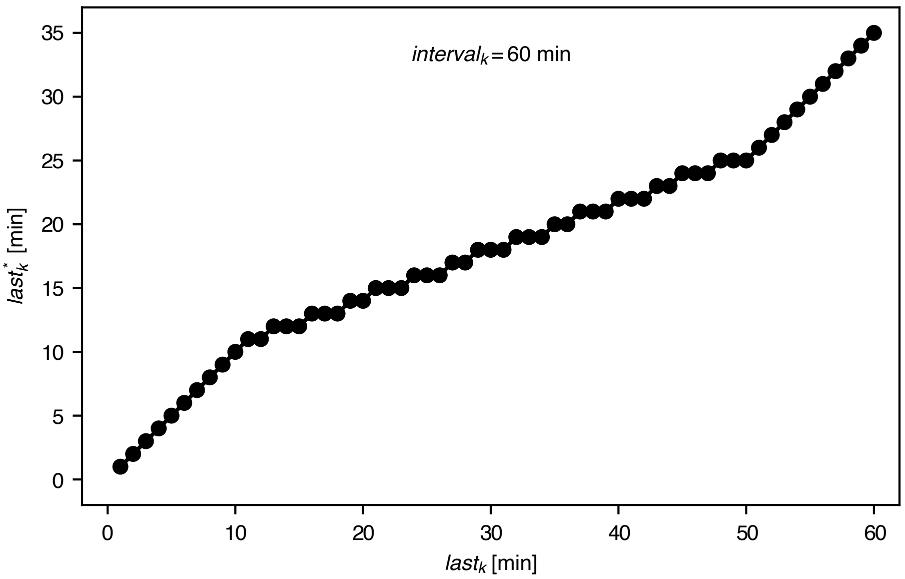

Fig S7. Imputation of responses after intervals not present in the dataset. Since we do not have experimental data for responses (*interval*$_k$, *last*$_k$) for *interval*$_k$ > 35 min, we instead sample from responses (*interval*$_k$* = 35 min, *last*$_k$*). The plot illustrates the mapping of *last*$_k$ to *last*$_k$* in the case of *interval*$_k$ = 60 min.

bioRxiv preprint doi: https://doi.org/10.1101/2025.04.30.651439; this version posted May 5, 2025. The copyright holder for this preprint (which was not certified by peer review) is the author/funder, who has granted bioRxiv a license to display the preprint in perpetuity. It is made available under aCC-BY 4.0 International license.

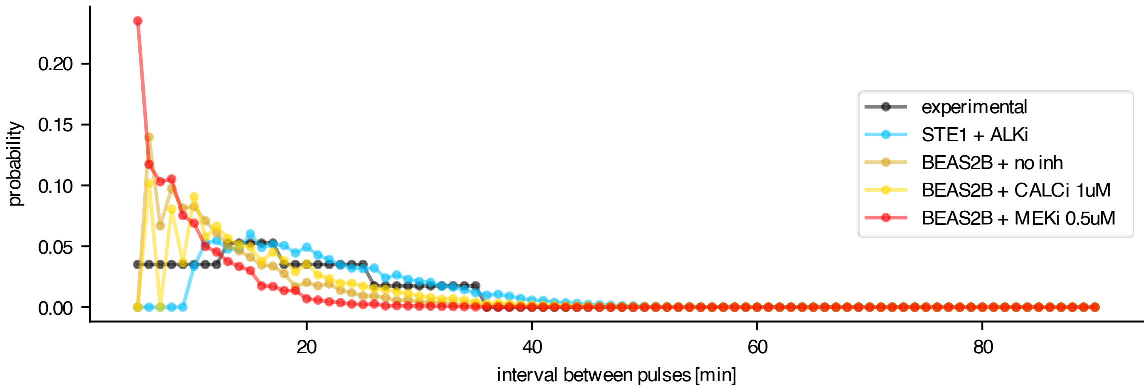

Fig S8. Input interval distribution optimized without regularization. Setup as in Fig 4C.
